# Supplementary material for: High Tau expression correlates with reduced invasion and prolonged survival in Ewing sarcoma
Source: Cell Death Discov. 2025 May 3;11:216. doi: 10.1038/s41420-025-02497-7 (PMC12049433; doi:10.1038/s41420-025-02497-7)
Supplement: Supplementary file 1 — Supplementary Figure Legends [file 41420_2025_2497_MOESM1_ESM.docx]

**Supplementary Figure Legends**

***Supplementary Figure 1. Effect of doxycycline-treatment of parental TC-32 cells on adhesion, migration, and invasion. A.*** *Lysates from biological triplicates were prepared from parental TC-32 cells not (ctrl) or treated (mock) with doxycycline for 2.5 weeks. Samples were analyzed by western blot with mouse Tau13 and rabbit GAPDH antibodies, followed by visualization with anti-mouse IgG IRDye 680RD and anti-rabbit IgG IRDye 800CW. Intensity of the signals is reported as ratio Tau/GAPDH (n=9).* ***B.*** *Parental TC-32 cells in MW96 were imaged for surface covered at the Incucyte every 2 h and cell proliferation calculated over 72 h starting from time 0 (n=12).* ***C.*** *Adherent parental TC-32 cells on the indicated cell substrates 30 min after plating and stained with crystal violet (OD590 nm normalized over ctrl (n= 4).* ***D.*** *TC-32 Tau-KD cells were seeded in transwell inserts without any coating (migration) or pre-coated with Matrigel* *(invasion). Cells were imaged on the other side of the filters 2 d later upon staining with crystal violet and quantified with ImageJ (n=3). Mock data were normalized over ctrl, reported as mean ± SD and analyzed with the Mann-Whitney test.*

***Supplementary Figure 2.*** ***Changes in P-FAK and vimentin are not artefacts of doxycycline treatment.*** *Lysates from biological triplicates were prepared from parental TC-32 not (ctrl) or treated (mock) or with doxycycline for 2.5 weeks. Samples were resolved on SDS-PAGE and analyzed by western blot with mouse P-FAK and rabbit FAK antibodies, the rabbit vimentin antibody, and the rabbit GAPDH antibody. Primary antibodies were detected using anti-rabbit IgG IRDye 800CW or anti-mouse IgG IRDye 680RD. Signal intensity was quantified and reported as ratio P-FAK/FAK or vimentin/GAPDH (n=9). Mock data were normalized over ctrl, reported as mean ± SD and analyzed with the Mann-Whitney test.*
