# Supplementary material for: High Tau expression correlates with reduced invasion and prolonged survival in Ewing sarcoma
Source: Cell Death Discov. 2025 May 3;11:216. doi: 10.1038/s41420-025-02497-7 (PMC12049433; doi:10.1038/s41420-025-02497-7)

## Slide 1
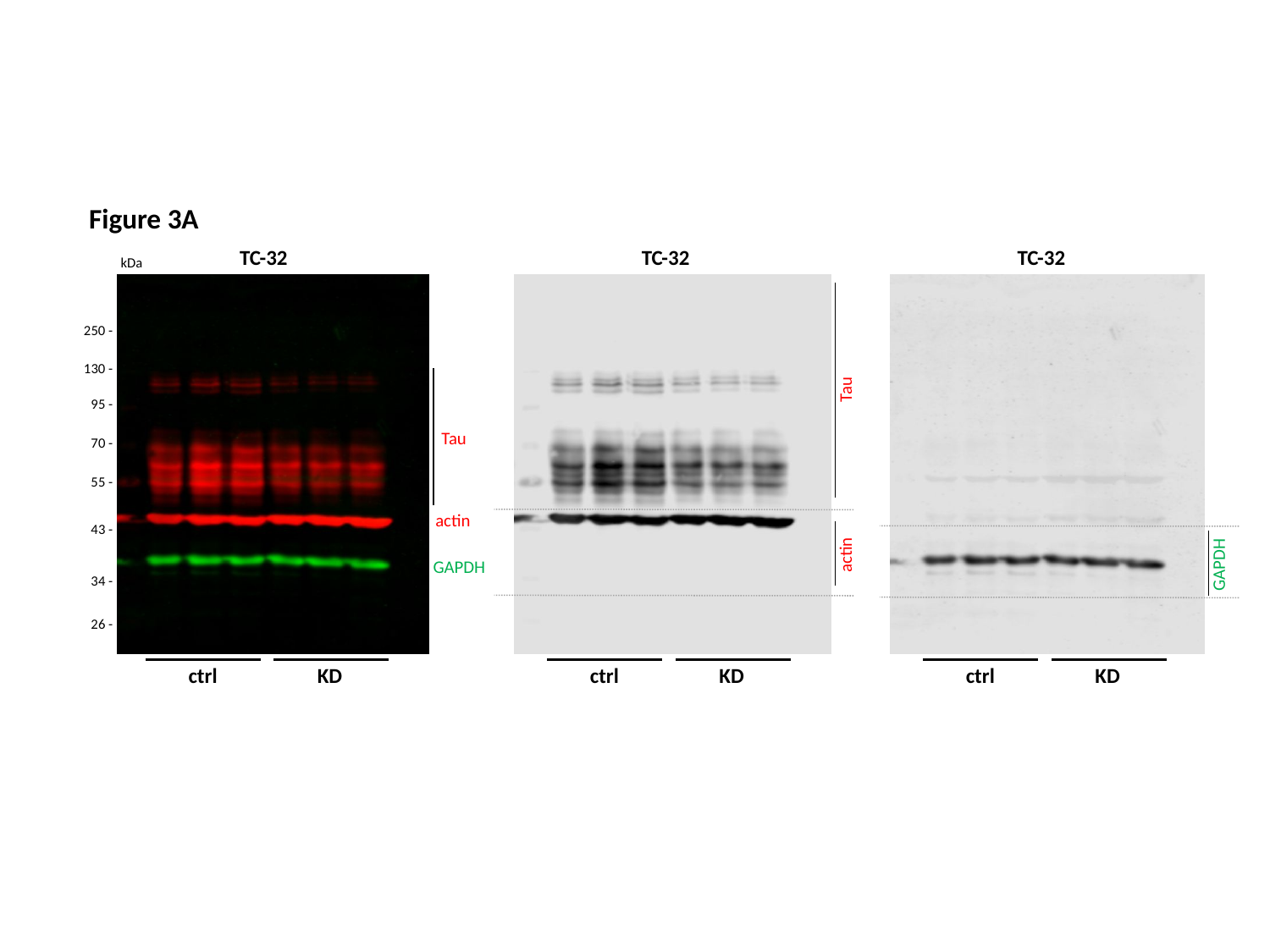

Figure 3A
TC-32
TC-32
TC-32
kDa
250 -
130 -
Tau
95 -
Tau
70 -
55 -
actin
43 -
actin
GAPDH
GAPDH
34 -
26 -
ctrl
KD
ctrl
KD
ctrl
KD

## Slide 2
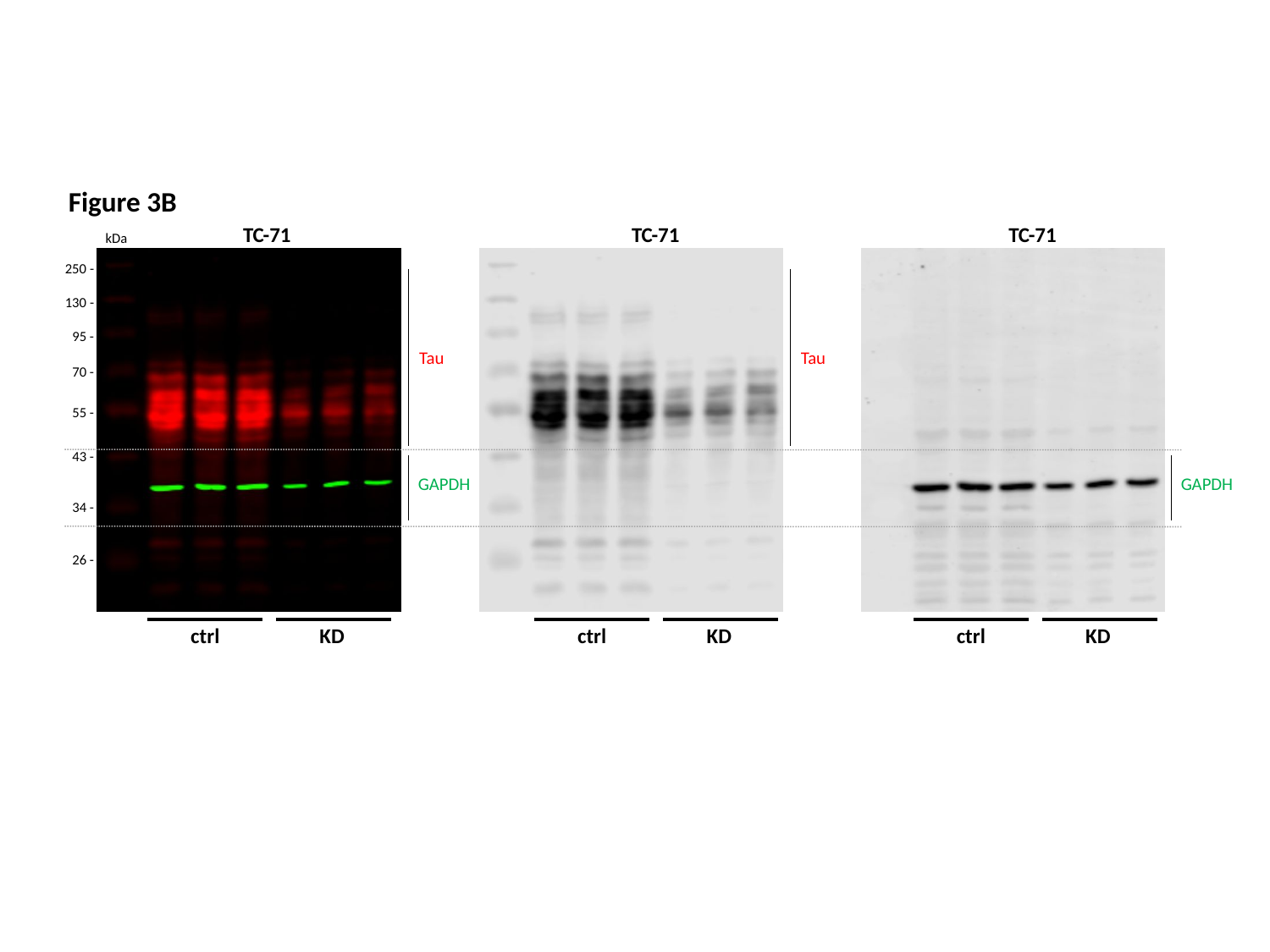

Figure 3B
TC-71
TC-71
TC-71
kDa
250 -
130 -
95 -
Tau
Tau
70 -
55 -
43 -
GAPDH
GAPDH
34 -
26 -
ctrl
KD
ctrl
KD
ctrl
KD

## Slide 3
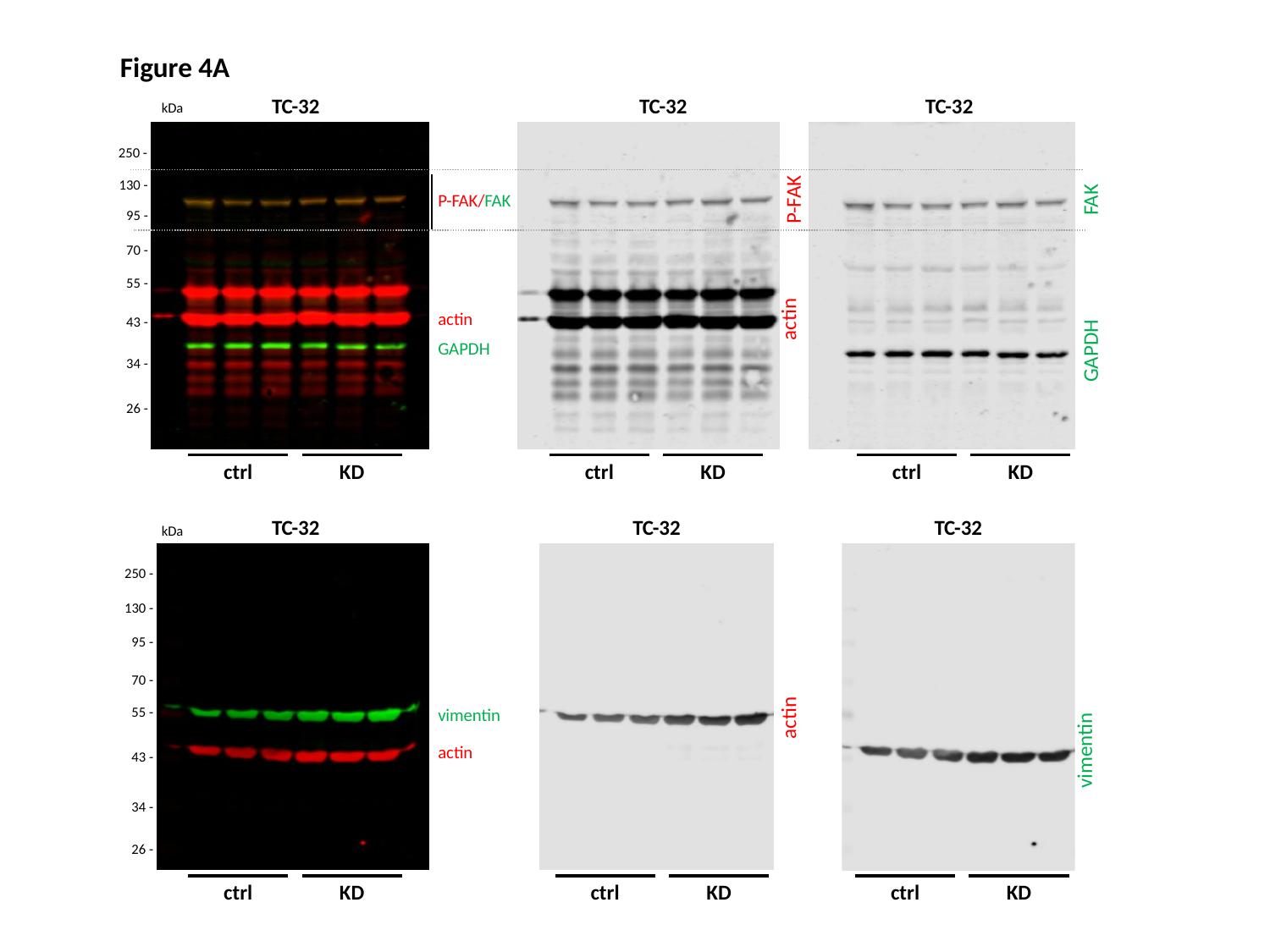

Figure 4A
TC-32
TC-32
TC-32
kDa
250 -
130 -
P-FAK
FAK
P-FAK/FAK
95 -
70 -
55 -
actin
actin
43 -
GAPDH
GAPDH
34 -
26 -
ctrl
KD
ctrl
KD
ctrl
KD
TC-32
TC-32
actin
ctrl
KD
TC-32
vimentin
ctrl
KD
kDa
250 -
130 -
95 -
70 -
55 -
vimentin
actin
43 -
34 -
26 -
ctrl
KD

## Slide 4
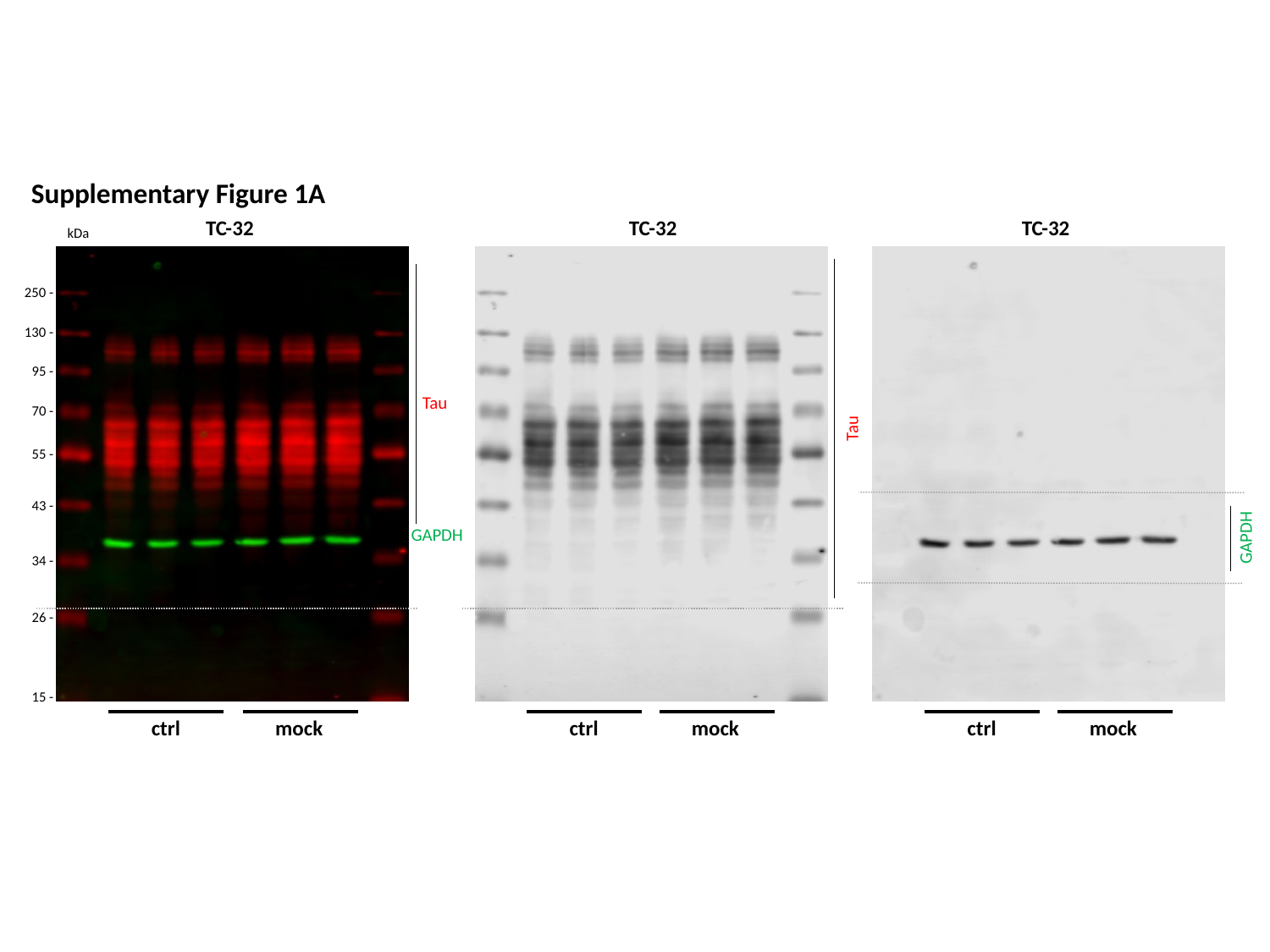

Supplementary Figure 1A
TC-32
kDa
kDa
250 -
130 -
95 -
Tau
70 -
55 -
43 -
GAPDH
34 -
26 -
15 -
ctrl
mock
TC-32
Tau
ctrl
mock
TC-32
GAPDH
ctrl
mock

## Slide 5
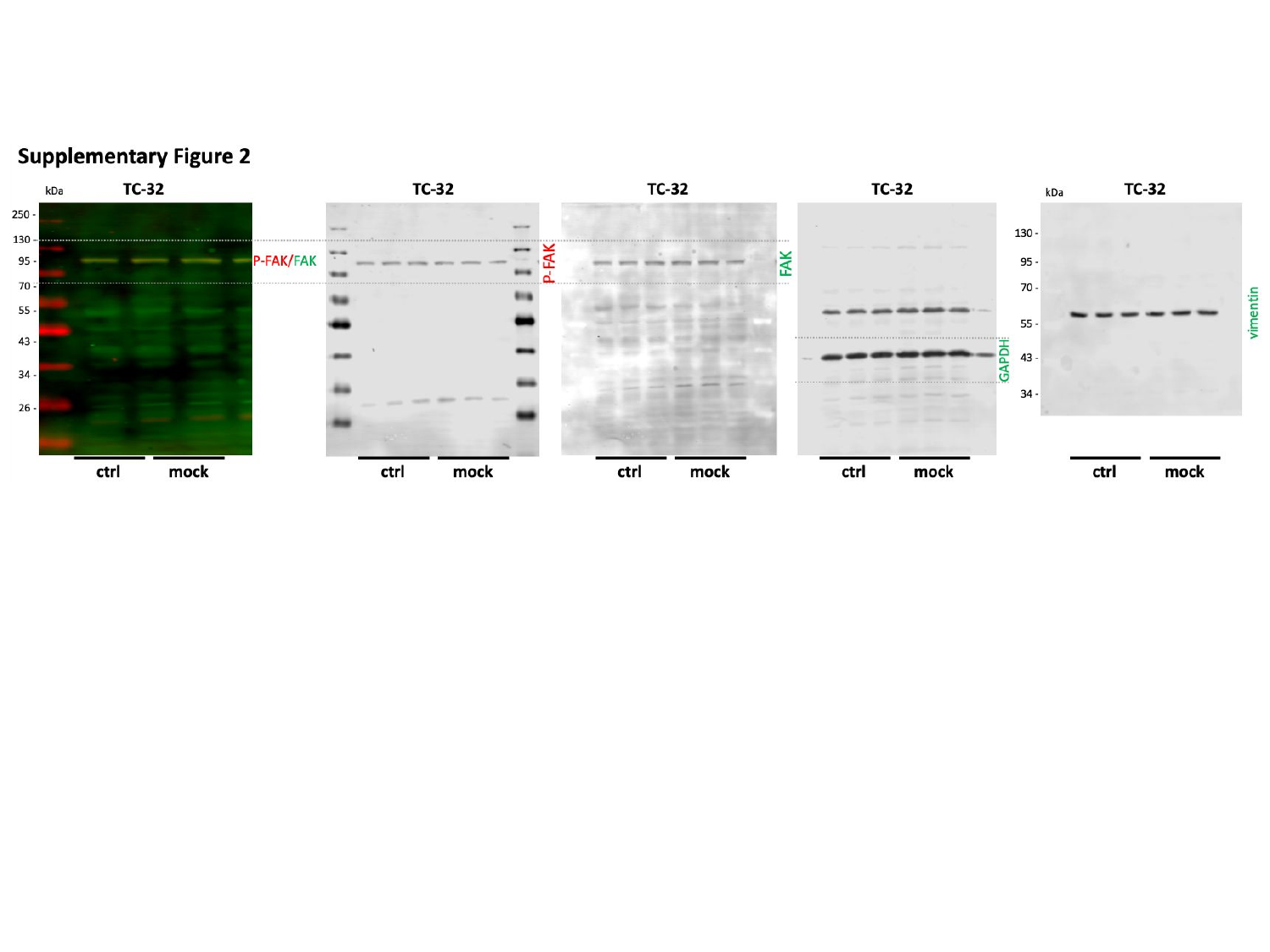

Supplement: Supplementary file 2 — Original data [file 41420_2025_2497_MOESM2_ESM.pptx]
