# Supplementary figures and images for: High Tau expression correlates with reduced invasion and prolonged survival in Ewing sarcoma
Source: Cell Death Discov. 2025 May 3;11:216. doi: 10.1038/s41420-025-02497-7 (PMC12049433; doi:10.1038/s41420-025-02497-7)

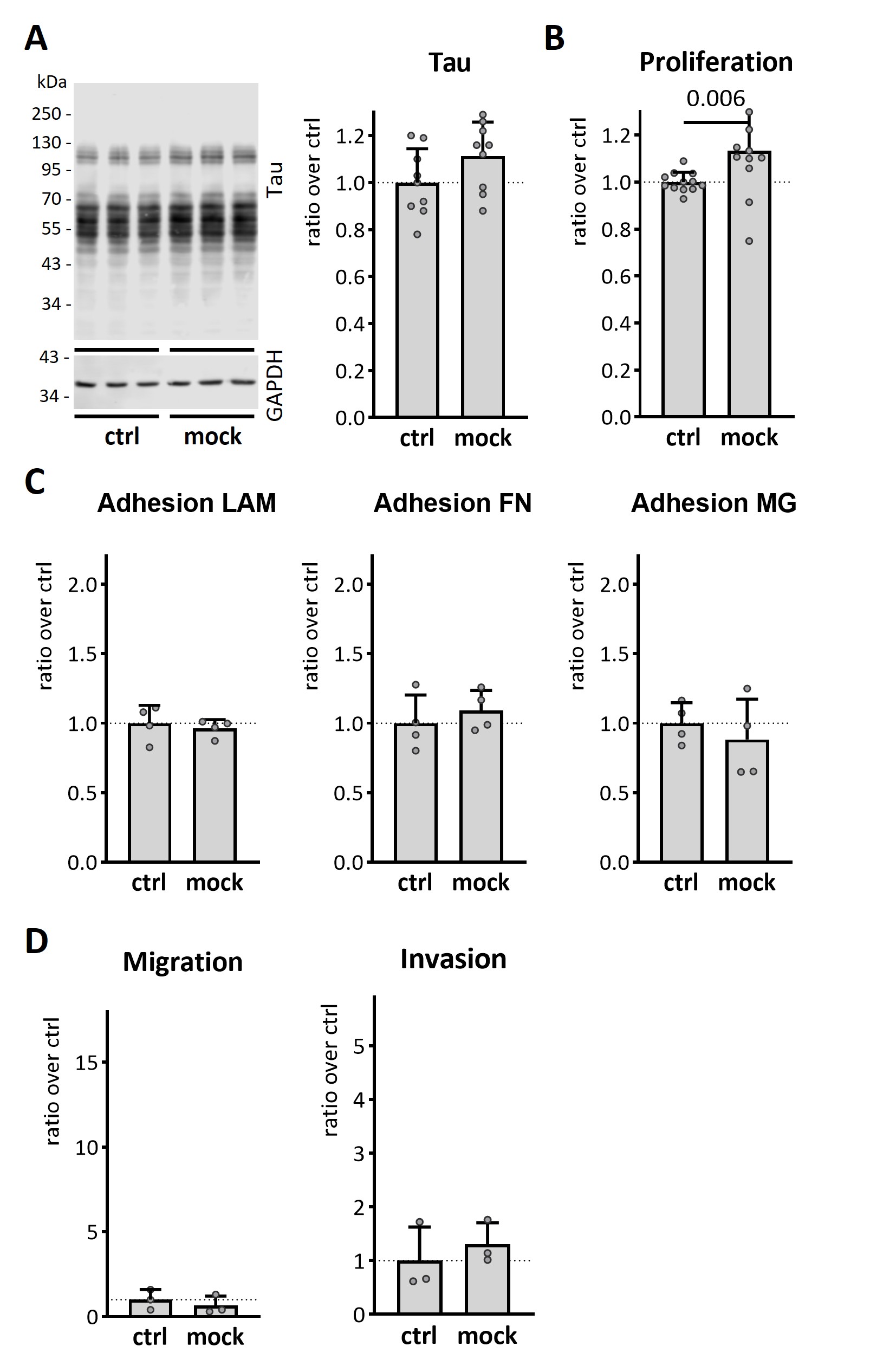

Supplement: Supplementary file 3 — Supplementary Figure 1 [file 41420_2025_2497_MOESM3_ESM.jpg]

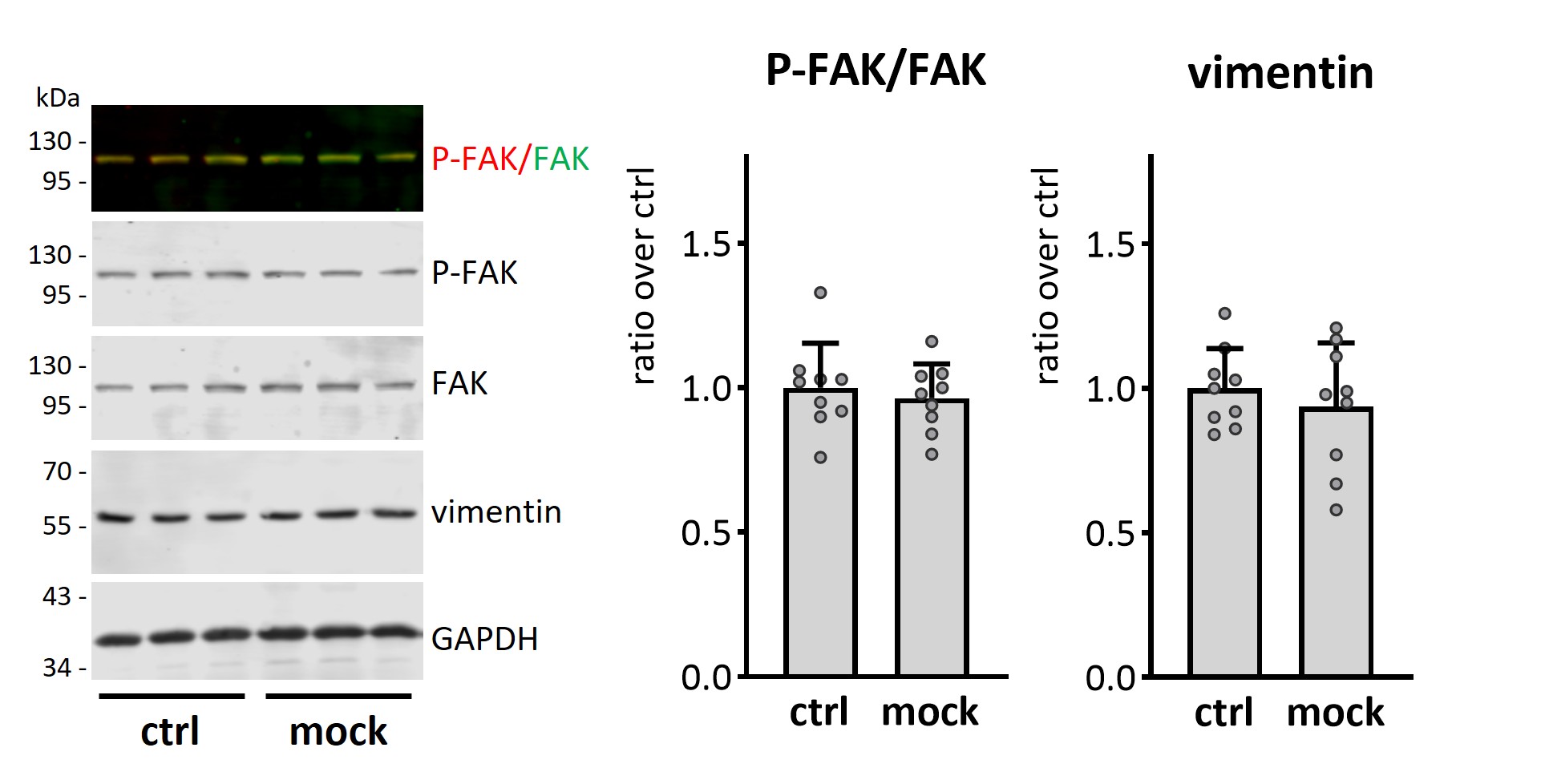

Supplement: Supplementary file 4 — Supplementary Figure 2 [file 41420_2025_2497_MOESM4_ESM.jpg]
